# Reply to "comment on: Transrectal prostate biopsy complications: a prospective single center study in a mid-income country"

**DOI:** 10.31744/einstein_journal/2026CE2377

**Published:** 2026-02-12

**Authors:** Allan Jefferson Schollemberg, Flavio Lobo Heldwein, Suelen dos Santos, Vítor Maurício Merlin Maschietto, Erick Schnorrenberger, Kleber Reinert, Gabriela Garcia Korczaguin, Marcelo Langer Wroclawski

**Affiliations:** 1 Hospital Governador Celso Ramos Florianópolis SC Brazil Hospital Governador Celso Ramos, Florianópolis, SC, Brazil.; 2 Universidade Federal de Santa Catarina Department of Urology Florianópolis SC Brazil Department of Urology, Universidade Federal de Santa Catarina, Florianópolis, SC, Brazil.; 3 Universidade do Sul de Santa Catarina Palhoça SC Brazil Universidade do Sul de Santa Catarina, Palhoça, SC, Brazil.; 4 Hospital Israelita Albert Einstein São Paulo SP Brazil Hospital Israelita Albert Einstein, São Paulo, SP, Brazil.; 5 A Beneficência Portuguesa de São Paulo Department of Urologic Oncology São Paulo SP Brazil Department of Urologic Oncology, A Beneficência Portuguesa de São Paulo, São Paulo, SP, Brazil.

Dear Editors,

We would like to thank Drs Hüsnü Tokgöz and Özlem Tokgöz for their thoughtful correspondence regarding our prospective cohort study on complications after transrectal ultrasound-guided prostate biopsy in a Brazilian public reference centre.^(1)^ In their letter, they accurately summarise our main findings, including a marked association between recent quinolone use and post-biopsy infectious complications.

Currently, the ongoing global struggle against bacterial resistance is a growing challenge to patient safety. Consequently, stewardship-driven approaches – such as monitoring local bacterial resistance patterns, adopting targeted prophylaxis (with different prophylactic regimens recommended by national and international agencies), and minimising unnecessary antibiotic exposure – are increasingly central to peri-procedural decision-making.^(2)^ In the specific context of prostate biopsy, a transperineal approach is recommended to minimise infectious complications.

It has been argued that recently published randomized studies may be underpowered to detect a rare yet feared complication such as urosepsis. Nevertheless, meta-analyses indicate a lower rate of this outcome and support the safety of the transperineal approach. (Table 1) Our research group has an ongoing meta-analysis protocol on this subject registered in PROSPERO (CRD42024556787), and we anticipate that the forthcoming synthesis will favour the transperineal approach regarding urosepsis (Figure 1).

**Table 1:** Meta-analysis of prospective and randomized clinical trials on urosepsis following prostate biopsy

Meta-analysis	Comparison	Urosepsis rate (%)			
		Transrectal	Transperineal		
			Periprocedural prophylactic antibiotics		
			With	Without	
Castellani et al, 2022^(3)^	TP with *versus w/o ATB prophylaxis*	-	0.13	0.09	RR: 1.09, (0.21-5.61), p=0.92
Wolff et al, 2024^(4)^	TP with *versus w/o ATB prophylaxis*	-	0.13	0.16	OR=1.3 (0.46-3.4), p=0.62
Madhavan et al, 2024^(5)^	TR *versus TP*	0.23	0.80		OR=0.49, (0.09–2.71), p=0.42
Zattoni et al, 2024^(6)^	TR *versus TP*	0.13	0		OR=0.6, (0.1–4.5)
Stangl et al, 2025^(7)^	TR *versus TP*	0.77	0.25		OR=0.49, (0.09–2.68) p=0.41
Our group (unpublished) PROSPERO CRD42024556787	TR *versus TP*	2.6	0.18		p<0.01 (excluding RCT with no urosepsis cases reported)

**Figure 1:**
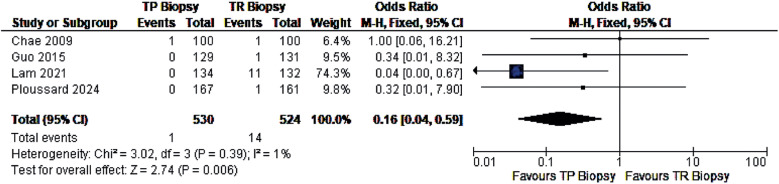
Forest-plot of urosepsis in randomized clinical trials comparing trasnrectal versus transperineal prostate biopsy

Source: Heldwein FL, Korczaguin GG, Santos S, Maschietto VMM. Transrectal versus transperineal prostate biopsy: a systematic review and meta-analysis of randomized controlled trials on complications and efficacy. PROSPERO. 2024 [cited 2025 Dec 07]. Available from: https://www.crd.york.ac.uk/PROSPERO/view/CRD42024556787^(8)^

## Data Availability

The content is already available.
